# Promoter Methylation Analysis of IDH Genes in Human Gliomas

**DOI:** 10.3389/fonc.2012.00193

**Published:** 2012-12-19

**Authors:** Simon Flanagan, Maggie Lee, Cheryl C. Y. Li, Catherine M. Suter, Michael E. Buckland

**Affiliations:** ^1^Discipline of Pathology, University of SydneySydney, NSW, Australia; ^2^Department of Neuropathology, Royal Prince Alfred HospitalSydney, NSW, Australia; ^3^Victor Chang Cardiac Research InstituteDarlinghurst, NSW, Australia

**Keywords:** isocitrate dehydrogenase, glioma, brain tumor, DNA methylation, pyrosequencing

## Abstract

Mutations in isocitrate dehydrogenase (IDH)-1 or -2 are found in the majority of WHO grade II and III astrocytomas and oligodendrogliomas, and secondary glioblastomas. Almost all described mutations are heterozygous missense mutations affecting a conserved arginine residue in the substrate binding site of IDH1 (R132) or IDH2 (R172). But the exact mechanism of IDH mutations in neoplasia is not understood. It has been proposed that IDH mutations impart a “toxic gain-of-function” to the mutant protein, however a dominant-negative effect of mutant IDH has also been described, implying that IDH may function as a tumor suppressor gene. As most, if not all, tumor suppressor genes are inactivated by epigenetic silencing, in a wide variety of tumors, we asked if *IDH1* or *IDH2* carry the epigenetic signature of a tumor suppressor by assessing cytosine methylation at their promoters. Methylation was quantified in 68 human brain tumors, including both IDH-mutant and IDH wildtype, by bisulfite pyrosequencing. In all tumors examined, CpG methylation levels were less than 8%. Our data demonstrate that inactivation of IDH function through promoter hypermethylation is not common in human gliomas and other brain tumors. These findings do not support a tumor suppressor role for IDH genes in human gliomas.

## Introduction

Mutations in isocitrate dehydrogenase (IDH)-1 or-2 are found in the majority of WHO grade II and III astrocytomas and oligodendrogliomas, and secondary glioblastomas (Parsons et al., 2008; Yan et al., 2009). IDH mutations are also found in some myeloid malignancies and chondroid tumors (Amary et al., 2011). IDH mutations occur early in gliomagenesis (Watanabe et al., 2009), suggesting that low grade diffuse gliomas share a common pathway in their development. Patients with high-grade gliomas carrying IDH mutations have significantly better survival than those with wildtype IDH tumors and also respond better to treatment (Parsons et al., 2008; Yan et al., 2009; Houillier et al., 2010). In addition, IDH mutation status is useful in assisting diagnostic classification: the majority of astrocytomas, oligodendrogliomas, and secondary glioblastomas harbor IDH mutations while almost all primary glioblastomas do not (reviewed in Gupta et al., 2011).

Isocitrate dehydrogenase is one of several citric acid cycle enzymes implicated in neoplasia. Mutations in all four subunits of succinate dehydrogenase (SDH) underlie a variety of sporadic and inherited paragangliomas and pheochromocytomas (Sudarshan et al., 2007), while mutations in fumarate hydratase are associated with hereditary leiomyomatosis and renal cell cancer and some cases of isolated type 2 papillary renal cell carcinoma (Gardie et al., 2011). SDH and fumarate hydratase are thought to act as tumor suppressor genes, with cancers exhibiting the “two-hits” typical of tumor suppressor gene inactivation (reviewed in Wallace, 2012).

Almost all described IDH mutations in gliomas are heterozygous missense mutations focused on a few conserved residues in the enzymes’ substrate binding sites. IDH mutations have thus been proposed to impart a toxic gain-of-function to the resultant protein (Dang et al., 2009). A salient biochemical feature of cells carrying mutant IDH is increased R-2 hydroxyglutarate (2HG) production via consumption of αKG and NADPH (Dang et al., 2009). But mature IDH1 and IDH2 enzymes are homodimeric, and it has also been demonstrated that mutant IDH1 exerts a dominant-negative effect on IDH function through mutant/wildtype heterodimer formation (Zhao et al., 2009); this scenario would lead to a direct effect on αKG and NADPH levels. We and others have previously reported loss of heterozygosity at the *IDH1* locus in gliomas and leukemias (Ichimura et al., 2009; Zhang et al., 2011; Gupta et al., 2012), and monoallelic expression of IDH1 in gliomas is not uncommon (Walker et al., 2012). Furthermore a recent report characterizes several rare but recurrent IDH mutations that result in loss-of-function without elevation of 2HG (Ward et al., 2012). Taken together, these findings suggest that at least in some circumstances *IDH1* and/or *IDH2* may function as a typical tumor suppressor gene.

As promoter hypermethylation is one hallmark of tumor suppressor genes in a variety of tumors (Baylin and Herman, 2000), we asked if IDH genes may carry this particular epigenetic signature of a tumor suppressor by assessing cytosine methylation at their respective promoters. Our study is the first to specifically examine IDH promoter methylation in tumors.

## Materials and Methods

### Tumors samples

Tumors were obtained from the Royal Prince Alfred Hospital tumor and tissue bank following appropriate institutional human research ethics approval. Histological diagnoses were provided by an experienced neuropathologist (Michael E. Buckland). The tumor samples included gliomas with a variety of *IDH1* mutations, as well as IDH-wildtype tumors (Table 1) and three samples of non-neoplastic brain. Also included in the group were two tumors with a proven *IDH1* mutation, but with absent staining by the IDH1 mutation-specific antibodies H09 and SMab-1 (see below). All other tumors with IDH1 R132H or R132S mutations showed positive immunostaining with H09 or SMab-1 antibodies, respectively.

**Table 1:** **Tumors tested, *IDH1* mutation status, and mean methylation levels**.

Diagnosis	*N*	IDH1 mutation	Mean IDH1 methylation (%)	Mean IDH2 methylation (%)
Glioblastoma	30	8	1.7 ± 0.9	1.7 ± 0.5
Astrocytoma	7	6	2.6 ± 0.8	3.0 ± 1.3
Oligodendroglioma	10	9	2.2 ± 0.7	2.1 ± 0.8
Pilocytic astrocytoma	2	0	2.1 ± 0.4	1.9 ± 0.0
Meningioma	16	0	2.3 ± 1.2	3.4 ± 1.6
Adenocarcinoma	3	0	2.2 ± 0.4	2.6 ± 0.9
Non-neoplastic brain	3	n/a	3.0 ± 1.75	3.9 ± 0.9

### Immunohistochemistry

Monoclonal antibodies against IDH1 R132H (clone H09; Dianova, Germany) and IDH1 R132S (kind gift from Dr. Y. Kato, Japan) were used at 1:500 dilution on 5 μm-FFPE tumor sections. Following antigen retrieval in 10 mM sodium citrate buffer pH 6.0, for 20 min at 125°C, sections were incubated in primary antibodies for 1 h at room temperature, and antibody detection was performed using the Dako Envision system, according to the manufacturer’s instructions.

### DNA extraction and bisulfite modification

DNA was extracted from 100 mg of frozen tumor tissue using the Qiagen DNeasy blood and tissue kit (Qiagen, Hilden, Germany), and bisulfite modification was performed using the Qiagen Epitect Bisulfite Kit (Qiagen, Hilden, Germany), according to the manufacturer’s instructions.

### Promoter methylation analysis

**Figure 1:**
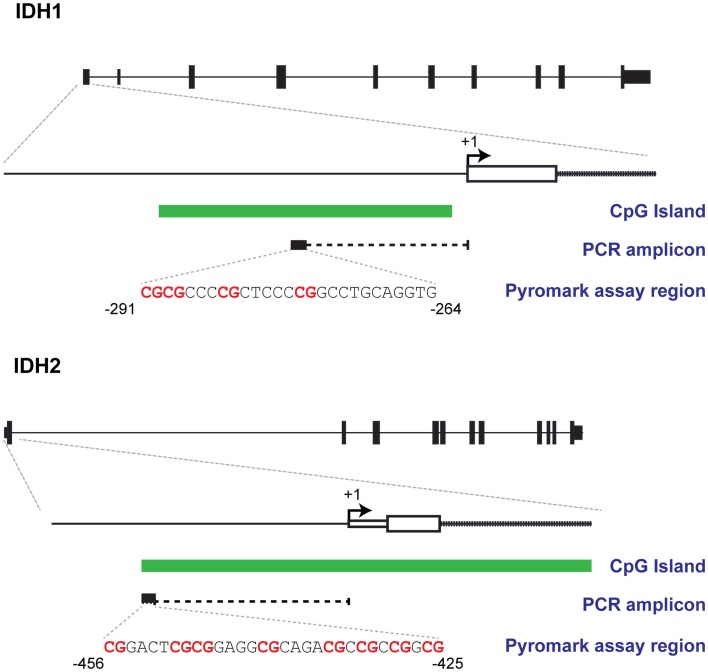
**Bisulphite Pyrosequencing designs**. Schematics showing regions targeted for methylation analysis and their relationships with CpG islands and transcription start sites of *IDH1* and *IDH2*.

Methylation status of the *IDH1* and *IDH2* promoter regions were assessed using Qiagen’s Pyromark CpG assays, Hs_IDH1_01_PM and Hs_IDH2_01_PM, respectively (see Figure 1). Pyrograms were analyzed using Pyromark Q24 software (Qiagen, Hilden, Germany), version 2.0.6, to calculate percentage methylation at each CpG and mean methylation across all CpGs for each sample was calculated.

### IDH1 and IDH2 mutation assessment

In tumors that were negative for IDH R132H immunostaining, *IDH1* and *IDH2* mutation status was determined by direct DNA sequencing. The fourth exons of *IDH1* and *IDH2* were PCR amplified in separate reactions using primer pairs CATTTGTCTGAAAAACTTTGCTT and TCACATTATTGCCAACATGAC for *IDH1*, and GGTTCAAATTCTGGTTGAAAGATG and GCTAGGCGAGGAGCTCCAGT for *IDH2*. Each reaction consisted of 50 mM Tris/HCl, 10 mM KCl, 5 mM (NH_4_)_2_SO_4_, 2 mM MgCl_2_, 0.2 mM each dNTP, 2 U FastStart Taq polymerase (Roche, Manheim, Germany), 0.5 μM each primer, and 2 μL of extracted DNA in a total volume of 20 μL. Cycling conditions were, initial denaturation at 95°C for 5 min, followed by 40 cycles of 95°C for 30 s, 60°C for 30 s, and 72°C for 30 s. PCR products were sequenced directionally using BigDye Terminator v3.1 Cycle Sequencing Kit (Applied Biosystems), and sequencing reaction products were resolved on a 3730xl DNA Analyzer (Applied Biosystems), according to the manufacturer’s instructions.

### Statistics

Two-tailed Student *t*-test was used to compare mean *IDH1* and *IDH2* promoter methylation levels between IDH-mutant and wildtype tumors.

## Results

**Figure 2:**
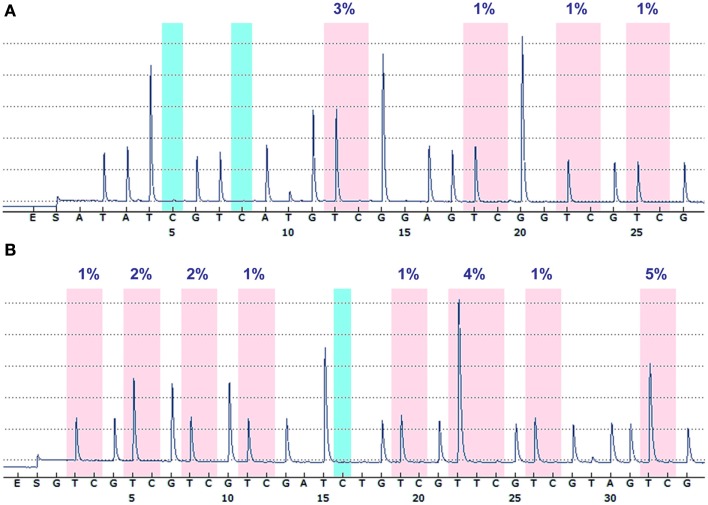
**Representative pyrograms**. Typical pyrograms obtained from patient samples for *IDH1* **(A)** and *IDH2* **(B)**. Each pink shaded column indicates a CpG dinucleotide assayed, and the percentage of methylation at that CpG dinucleotide is indicated above. The blue shaded boxes are internal bisulfite modification control assessments.

Figure 1 shows the promoter regions of *IDH1* and *IDH2*, the CpG island within these regions, and the sequences targeted for bisulfite pyrosequencing. The *IDH1* assay targets four contiguous CpG sites, 275 bp upstream of the transcription start site. The *IDH2* assay targets eight CpG sites 425 bp upstream from the transcription start site. The CpGs targeted by these assays lie within CpG islands that are adjacent to, or span, the transcription start site of the gene. Typical pyrograms obtained for patient samples for both *IDH1* and *IDH2* assays are shown in Figure 2.

A total of 68 brain tumors were assessed for *IDH1* and *IDH2* promoter methylation status. Tumors examined consisted of 49 gliomas, 16 meningiomas, and 3 metastatic adenocarcinomas. Twenty five *IDH1* mutations were present in the gliomas tested (23 × R132H, 1 × R132S, and 1 × R132L); no *IDH2*-mutant tumors were present. Two of the mutant tumors (1 × R132H and 1 × R132S) were consistently negative for mutant protein expression on immunohistochemistry; all other R132H-mutant tumors were immunopositive with H09 antibody. Table 1 shows the number of tumor types examined, their *IDH1* mutation status, and mean promoter methylation levels for *IDH1* and *IDH2*. Table A1 in Appendix details the tumor type, WHO grade, IDH mutation status, and the methylation levels at each individual CpG dinucleotide for all samples included in this study.

For *IDH1*, mean of methylation across all tumors was 2% (below the reported detection limit of pyrosequencing; Mikeska et al., 2011), with average methylation across the four CpGs assayed for each sample ranging from 0.3 to 5.3%. The highest methylation level detected (5.3%) was in a secondary glioblastoma that harbored an IDH1 R132H mutation. No other samples in the study showed methylation above 5% at the *IDH1* promoter (Table A1 in Appendix). The three non-neoplastic brain samples had an average methylation level of 3%. There was no significant difference in mean methylation levels between wildtype tumors and those harboring an *IDH1* mutation.

For *IDH2*, average methylation across all tumor samples was 2.3%, and the highest average methylation across the eight CpGs assayed shown by any sample was 7.1% in a meningioma (Table A1 in Appendix). Three non-neoplastic control brain samples had an average methylation level of 3.9%. Only one other sample, also a meningioma, recorded an average methylation at the *IDH2* promoter above 5%. *In vitro* methylated genomic DNA (Qiagen, Hilden, Germany) showed average methylation of 85.2 and 87.7% for the *IDH1* and *IDH2* loci, respectively, confirming assay performance.

Two gliomas in our cohort were found to harbor *IDH1* mutations when subjected to DNA sequencing, but exhibited no protein expression by immunohistochemistry, despite repeated testing. Following negative immunohistochemistry results, we were able to extract DNA from the slide used for immunostaining, and confirm the presence of *IDH1* mutation, excluding tissue mosaicism as a cause of these results. The two IDH-mutant tumors without detectable mutant protein expression showed mean IDH1 methylation levels of 2.8% (R132H), and 1.8% (R132S) only.

## Discussion

In this study, we quantified methylation levels within the CpG island associated with the promoter regions of both *IDH1* and *IDH2* by bisulfite pyrosequencing. We hypothesized that if *IDH1* and *IDH2* functioned as tumor suppressor genes, then some tumors may exhibit dense hypermethylation of the CpG island associated with their promoter region. Loss of gene expression associated with promoter methylation is a well described phenomenon in a variety of tumors (Baylin and Herman, 2000). However, none of the 68 tumors examined exhibited this epigenetic hallmark of tumor suppressor genes.

Three IDH enzymes are present in mammalian cells, IDH1, IDH2, and IDH3. IDH3 utilizes NAD+ as the reducing agent in an irreversible reaction to convert mitochondrial isocitrate to αKG, while both IDH1 and IDH2 utilize NADP+ to convert isocitrate to αKG in a reversible fashion. To date, *IDH3* mutations have not been implicated in neoplasia. Mutant IDH1 and IDH2 enzymes exert a novel biochemical effect, namely the production of 2HG, via consumption of αKG and NADPH (Dang et al., 2009). 2HG is a competitive inhibitor of αKG-dependant enzymes such as histone lysine methyltransferases and the TET family of 5-methylcytosine hydroxylases. Both reduced αKG and increased 2HG levels converge to alter the cellular epigenetic landscape; *in vitro* transfection of mutant IDH into immortalized astrocytes results in increased histone methylation and a gradual rise in DNA methylation levels (Lu et al., 2012). In addition, the recently described glioma CpG island hypermethylator phenotype is associated with IDH mutations (Turcan et al., 2012).

*IDH1* is also the major source of non-mitochondrial NADPH in humans, which is important in the control of oxidative damage. Either loss-of-function or gain-of-function of IDH will result in reduced cytoplasmic NADPH levels and susceptibility to oxidative stress (Reitman and Yan, 2010). It may be that neomorphic production of 2HG and decreased enzymatic activity are both involved in tumor development, but in different contexts. In support of this, rare IDH mutations in lymphoma, pediatric glioblastoma, and thyroid cancer have demonstrated loss-of-function effects (Ward et al., 2012). Assessment of IDH promoter methylation in these tumor types may assist in determining if tissue-specific roles exist for IDH mutations in cancer.

Monoallelic expression of *IDH1* has recently been reported in 12 out of 67 gliomas with *IDH1* mutations (Walker et al., 2012), with expression occurring only from the wildtype allele in 10 of 12 cases. Preferential or exclusive expression of the wildtype *IDH1* allele was associated with significantly poorer outcomes (Walker et al., 2012). Included in our cohort were two tumors harboring *IDH1* mutations but without detectable mutant protein expression by immunohistochemistry, raising the possibility of monoallelic expression from the wildtype allele. However, neither of these tumors showed significant promoter methylation, excluding this epigenetic regulatory mechanism as the cause of undetectable mutant protein. Further studies into the mechanism(s) involved in monoallelic expression and its role in progression of glioma and other cancers will provide additional insights into the role of IDH in neoplasia.

Molecular testing for IDH mutations is useful in the clinical setting in assisting diagnostic classification and as a prognostic indicator. While there is a routinely used, specific antibody for IDH1 R132H mutations, it is becoming common to screen tumors with negative IDH immunostaining using DNA based techniques. The identification of monoallelic expression of *IDH1* in glioma raises potential issues in the clinical interpretation of these molecular analyses. It may be that monoallelic (or even skewed) gene expression could compromise the prognostic utility of DNA testing for a subset of tumors.

In this study, we have demonstrated that promoter hypermethylation of *IDH1* and *IDH2* genes is absent or very rare in human gliomas and other brain tumors. These findings do not support a tumor suppressor gene mechanism for IDH in human gliomas. However the occurrence of uncommon inactivating IDH mutations, loss of heterozygosity of IDH in some tumors, and monoallelic expression of *IDH1* in some gliomas points to as yet uncharacterized mechanisms of disease associated with IDH dysfunction.

## Conflict of Interest Statement

The authors declare that the research was conducted in the absence of any commercial or financial relationships that could be construed as a potential conflict of interest.

## Appendix

**Table A1:** **Detailed results of tumours tested**.

Tumor type	Grade	Mutation status	IDH1 methylation (%)					IDH2 methylation (%)								
			CPG1	CPG2	CPG3	CPG4	AVG	CPG1	CPG2	CPG3	CPG4	CPG5	CPG6	CPG7	CPG8	AVG
Astrocytoma	III	R132H	3	1	1	1	1.5	2	2	1	1	2	4	1	4	2.1
Astrocytoma	II	R132H	4	1	1	1	1.8	1	2	1	1	1	4	1	4	1.9
Astrocytoma	III	R132H	6	3	3	3	3.8	4	5	3	3	2	8	2	6	4.1
Astrocytoma	III	R132H	6	3	3	3	3	3	5	3	2	2	9	2	7	4.1
Glioblastoma	IV	R132H	4	3	5	9	5.3	2	3	1	1	1	4	1	2	1.9
Glioblastoma	IV	R132H	2	1	1	1	1.3	1	1	1	1	1	2	1	2	1.3
Glioblastoma	IV	R132H	2	1	1	1	1	1	0	0	0	1	3	1	5	1.4
Glioblastoma	IV	R132H	1	1	1	1	1	1	1	0	0	1	3	1	3	1.3
Glioblastoma	IV	R132H	2	1	1	1	1.3	1	1	1	0	2	3	1	6	1.9
Glioblastoma	IV	R132H	2	1	1	1	1.3	1	1	1	0	1	3	1	5	1.6
Glioblastoma	IV	R132H	2	1	1	1	1.3	0	1	0	0	0	2	0	2	0.6
Oligodendroglioma	III	R132H	4	1	1	1	1.8	1	2	1	1	1	3	1	3	1.6
Oligodendroglioma	III	R132H	4	1	2	1	2	1	2	1	1	1	4	1	4	1.9
Oligodendroglioma	III	R132H	4	2	2	1	2.3	3	2	1	1	1	4	1	3	2
Oligodendroglioma	III	R132H	4	2	2	1	2.3	1	3	1	1	1	4	1	3	1.9
Oligodendroglioma	III	R132H	4	2	1	1	2	1	2	1	1	1	4	1	3	1.8
Oligodendroglioma	III	R132H	4	2	2	1	2.3	2	3	1	1	1	5	1	3	2.1
Oligodendroglioma	II	R132H	4	1	1	1	1.8	1	2	1	1	1	5	1	4	2
Oligodendroglioma	II	R132H	3	1	1	1	1.5	1	2	1	1	1	5	1	2	1.8
Oligodendroglioma	II	R132H	4	2	1	1	2	1	2	1	1	1	4	1	3	1.8
Oligodendroglioma	III	R132H	7	3	3	3	4	4	6	3	3	3	9	2	6	4.5
Astrocytoma*	III	R132H*	5	2	2	2	2.8	2	4	2	3	4	7	2	4	3.5
Astrocytoma	III	R132L	4	2	2	1	2.3	2	3	2	2	6	7	2	11	4.4
Glioblastoma*	IV	R132S*	2	1	4	0	1.8	2	2	1	1	1	3	1	3	1.8
Adenocarcinoma	N/A	Wildtype	4	2	2	1	2.3	1	2	1	1	2	5	1	4	2.1
Adenocarcinoma	N/A	Wildtype	3	2	1	1	1.8	2	2	1	1	1	5	1	4	2.1
Adenocarcinoma	N/A	Wildtype	4	2	2	2	2.5	3	3	2	2	2	7	2	8	3.6
Astrocytoma	III	Wildtype	6	2	2	2	3	0	0	0	0	0	6	0	3	1.1
Glioblastoma	IV	Wildtype	4	2	2	1	2.3	2	2	2	2	1	4	1	3	2.1
Glioblastoma	IV	Wildtype	4	2	3	3	3	2	2	1	1	2	5	1	3	2.1
Glioblastoma	IV	Wildtype	4	1	0	0	1.3	2	2	0	1	1	4	0	3	1.6
Glioblastoma	IV	Wildtype	4	2	2	2	2.5	2	2	1	1	2	4	1	5	2.3
Glioblastoma	IV	Wildtype	3	2	2	2	2.3	2	3	2	1	1	4	1	4	2.3
Glioblastoma	IV	Wildtype	1	1	1	2	1.3	2	2	1	1	1	2	1	3	1.6
Glioblastoma	IV	Wildtype	2	1	1	1	1.3	1	2	2	1	2	3	1	8	2.5
Glioblastoma	IV	Wildtype	1	0	0	0	0.3	1	1	1	1	1	2	1	3	1.4
Glioblastoma	IV	Wildtype	3	2	2	2	2.3	2	2	1	1	0	5	0	0	1.4
Glioblastoma	IV	Wildtype	3	1	1	1	1.5	2	2	1	2	1	4	1	3	2
Glioblastoma	IV	Wildtype	3	2	3	3	2.8	2	2	1	1	1	4	1	4	2
Glioblastoma	IV	Wildtype	3	1	1	1	1.5	3	3	2	2	2	4	1	4	2.6
Glioblastoma	IV	Wildtype	2	1	1	1	1.3	1	2	1	1	1	4	1	4	1.9
Glioblastoma	IV	Wildtype	3	1	2	2	2	2	2	1	1	1	3	1	3	1.8
Glioblastoma	IV	Wildtype	2	1	1	1	1.3	1	2	1	1	1	3	1	3	1.6
Glioblastoma	IV	Wildtype	1	0	1	1	0.8	0	0	0	0	1	0	0	4	0.6
Glioblastoma	IV	Wildtype	3	1	1	1	1.5	0	1	0	0	1	3	0	2	0.9
Glioblastoma	IV	Wildtype	1	0	1	1	0.8	1	1	0	1	2	3	1	3	1.5
Glioblastoma	IV	Wildtype	2	1	1	1	1.3	0	0	0	0	1	3	0	5	1.1
Glioblastoma	IV	Wildtype	2	1	1	1	1.3	1	2	1	1	1	4	1	4	1.9
Glioblastoma	IV	Wildtype	2	1	1	1	1.3	1	1	1	1	2	3	1	3	1.6
Glioblastoma	IV	Wildtype	2	1	1	1	1.3	0	1	0	0	1	3	1	2	1
Meningioma	I	Wildtype	4	1	2	1	2	3	2	3	2	5	8	4	30	7.1
Meningioma	I	Wildtype	3	2	1	1	1.8	2	2	2	1	4	7	2	12	4
Meningioma	I	Wildtype	4	2	1	1	2	2	3	1	1	2	4	1	5	2.4
Meningioma	I	Wildtype	3	1	1	1	1.5	1	2	1	1	1	5	1	4	2
Meningioma	I	Wildtype	4	2	2	1	2.3	1	3	1	1	2	5	1	5	2.4
Meningioma	I	Wildtype	2	1	0	0	0.8	1	1	1	1	1	4	1	8	2.3
Meningioma	I	Wildtype	2	1	1	1	1.3	1	2	1	1	1	3	1	4	1.8
Meningioma	I	Wildtype	2	1	1	0	1	2	2	2	2	4	7	3	12	4.3
Meningioma	II	Wildtype	3	1	1	1	1.5	1	3	0	1	1	4	1	5	2
Meningioma	I	Wildtype	2	1	1	1	1.3	1	2	2	1	1	4	1	5	2.1
Meningioma	I	Wildtype	2	1	1	1	1.3	0	1	1	0	1	3	0	4	1.3
Meningioma	I	Wildtype	6	3	3	3	3.8	3	6	3	3	3	9	2	8	4.6
Meningioma	II	Wildtype	7	3	4	2	4	4	6	3	3	2	10	3	8	4.9
Meningioma	I	Wildtype	7	3	3	3	4	3	6	3	2	3	9	2	9	4.6
Meningioma	II	Wildtype	7	3	3	3	4	4	5	3	3	3	10	3	11	5.3
Meningioma	I	Wildtype	5	3	3	3	3.5	2	4	2	2	2	8	1	7	3.5
Pilocytic astrocytoma	I	Wildtype	4	2	2	1	2.3	2	2	1	1	1	4	1	3	1.9
Pilocytic astrocytoma	I	Wildtype	3	1	2	1	1.8	1	1	2	1	1	4	1	4	1.9
*t*-Test WTvs MUT (all tumors)			0.155	0.230	0.162	0.242	0.194	0.448	0.319	0.387	0.391	0.431	0.414	0.436	0.090	0.298

*Methylation is reported as a percentage of methylated alleles. Mutated tumours with absent mutant protein are marked by **.
